# Three-Phase Six-Level Multilevel Voltage Source Inverter: Modeling and Experimental Validation

**DOI:** 10.3390/mi12091133

**Published:** 2021-09-21

**Authors:** Sheikh Tanzim Meraj, Nor Zaihar Yahaya, Kamrul Hasan, Molla Shahadat Hossain Lipu, Ammar Masaoud, Sawal Hamid Md Ali, Aini Hussain, Muhammad Murtadha Othman, Farhan Mumtaz

**Affiliations:** 1 Department of Electrical and Electronic Engineering, Universiti Teknologi PETRONAS, Seri Iskandar 32610, Perak, Malaysia; sheikh_19001724@utp.edu.my (S.T.M.); norzaihar_yahaya@utp.edu.my (N.Z.Y.); fahan_19001785@utp.edu.my (F.M.); 2 Solar Research Institute (SRI), Universiti Teknologi MARA, Shah Alam 40450, Selangor, Malaysia; 2019984679@isiswa.uitm.edu.my (K.H.); m_murtadha@uitm.edu.my (M.M.O.); 3 Department of Electrical, Electronic and Systems Engineering, Universiti Kebangsaan Malaysia, UKM Bangi 43600, Selangor, Malaysia; sawal@ukm.edu.my (S.H.M.A.); draini@ukm.edu.my (A.H.); 4 Centre for Automotive Research (CAR), Universiti Kebangsaan Malaysia, UKM Bangi 43600, Selangor, Malaysia; 5 Faculty of Mechanical and Electrical Engineering, Al Baath University, Homs 22743, Syria; ammarshz123@gmail.com

**Keywords:** multilevel inverter, power electronics, staircase modulation, vector modulation

## Abstract

This research proposes a three-phase six-level multilevel inverter depending on twelve-switch three-phase Bridge and multilevel DC-link. The proposed architecture increases the number of voltage levels with less power components than conventional inverters such as the flying capacitor, cascaded H-bridge, diode-clamped and other recently established multilevel inverter topologies. The multilevel DC-link circuit is constructed by connecting three distinct DC voltage supplies, such as single DC supply, half-bridge and full-bridge cells. The purpose of both full-bridge and half-bridge cells is to provide a variable DC voltage with a common voltage step to the three-phase bridge’s mid-point. A vector modulation technique is also employed to achieve the desired output voltage waveforms. The proposed inverter can operate as a six-level or two-level inverter, depending on the magnitude of the modulation indexes. To guarantee the feasibility of the proposed configuration, the proposed inverter’s prototype is developed, and the experimental results are provided. The proposed inverter showed good performance with high efficiency of 97.59% following the IEEE 1547 standard. The current harmonics of the proposed inverter was also minimized to only 5.8%.

## 1. Introduction

In the sector of power engineering, multilevel inverters (MLIs) have been developed and their applications have been expanded in a rapid manner in recent years. The composition of MLIs basically includes a power components’ array and DC voltage supplies/capacitors that produce a variable and controllable stepped voltage waveform. Over the years, various MLIs topologies have been proposed and used due to their importance and roles in power conversion systems, with some of the most well-known and conventional topologies including the flying capacitor (FC), the cascaded H-bridge (CHB) and the neutral point clamped (NPC) inverter. Compared to the conventional two-level inverter, the CHB, FC and NPC multilevel inverters exhibit several advantageous features such as low voltage stress, low total harmonic distortions (THD), low switching losses, high electro-magnetic compatibility and high-quality output voltage with high efficiency [1,2,3,4].

To date, MLIs have been extensively used to control variable frequency drivers [5], static VAR compensators [6,7], HVDC systems [8], un-interrupted power supplies (UPS) and PV grid connected systems and renewable energy resources. By increasing the resolution of stepped voltage, the power quality of MLIs can be significantly improved. However, this process can significantly increase the number of power electric components count, installation area along with increased cost and control complications. Thus, the increased cost of the system and complexity of the inverter generally can limit the amount of voltage levels produced by any MLI structure.

To mitigate and balance out this particular issue, many variations of MLI topologies have been proposed in recent years to enhance their capability and utilize them in various industrial applications. Advanced NPC (ANPC) [9], the stacked MC [10], symmetrical and asymmetrical CHB are some notable examples [11,12,13,14]. Symmetrical MLIs utilize DC voltage sources of equal magnitudes and can have certain beneficial features such as, standardized control, lower voltage stresses on semiconductor devices, modular structure, etc. Hybrid MLIs are some alternative inverters that are built using the combination of two or more MLI topologies. These inverters are generally designed using DC supplies of unequal magnitudes. These MLIs can generate higher voltage levels utilizing less amount of power equipment and using a high voltage ratio of DC supplies [15,16]. Although asymmetry and hybridization can increase the number of voltage levels, these MLI topologies usually generate a high total standing voltage (TSV) on power electric components which in turn can increase the overall system cost, increase the voltage ratings of switches, decrease the switching redundancies and also limit the industrial applications. However, in [17], a novel family of MLI structures was designed using half-bridge inverter and multilevel DC-link. These variations in inverter topologies may produce even higher voltage levels utilizing abridged amount of power components. 

The goal of the multilevel DC-link is to provide the H-bridge with successive positive voltage levels starting from zero voltage, whereas the purpose of the half-bridge is to provide the desired outputs comprising of negative and positive voltage. The aforementioned method has been utilized to propose different three-phase cascaded MLI topologies which are presented in [18,19,20]. To further decrease the overall amount of DC supplies and switches in these topologies, a new multilevel inverter configuration has been recently suggested in [21,22,23,24]. These MLIs were built by utilizing the combination of a twelve-switch three-phase Bridge and a multilevel DC-link. The multilevel DC-link is constructed by a fixed supply of cascaded H-bridge power modules and DC voltage. Comparing with the available MLI topologies, the projected configuration comes with more voltage levels and lesser power components. 

In this study, an improved MLI is configured that can synthesize six-level output by using the combination of a conventional twelve-switch three-phase bridge and a modified multilevel DC-link. The multilevel DC-link is composed of only one DC voltage supply, H-bridge and full-bridge power cells. Both of the H-bridge and full-bridge modules are controlled, and the proposed inverter generates a stair-case waveform of eleven symmetrical line-to-line voltage levels. The proposed inverter needs to be operated under a certain condition where the modulation index is more than 0.98 to generate 11 voltage levels. On the other hand, the inverter can generate a stair-case waveform of nine asymmetrical lines to line voltage levels if the modulation index is equal to 0.98. Furthermore, if the modulation index decreases below 0.98, the MLI operates identically to a traditional two-level three-phase inverter and accordingly three symmetrical voltage levels are seen in its line-to-line output voltage waveforms. This research offers the following key contributions:

- The advantages of both multilevel DC-links and H-bridge circuits have been inherited by the suggested topology. The proposed MLI has been able to lower total voltage stress and generate greater voltage levels with fewer components because of this smart design.
- Since it does not contain switched capacitors, the proposed MLI does not require a complex control approach or additional circuits to deal with voltage balancing or excessive power losses.
- Depending on the application needs, the proposed topology can be modified to achieve higher voltage levels.
- The suggested MLI’s modularity and smart switching arrangements have allowed it to perform in four different ways depending on the modulation indices. This has provided the MLI with more flexibility and reliability.

The paper is organized into 6 sections. The MLI topology and its operation are studied in Section 2. Section 2 further discusses the efficiency, extended version of the MLI and the implementation of vector modulation technique. Section 3 shows the detailed simulation results of the proposed MLI. The comparison of this MLI with classical and recent MLI topologies is discussed in Section 4. Section 5 contains the experimental results, and Section 6 concludes the paper.

## 2. Operating Principle of Three-Phase MLI

### 2.1. Proposed Topology

**Figure 1:**
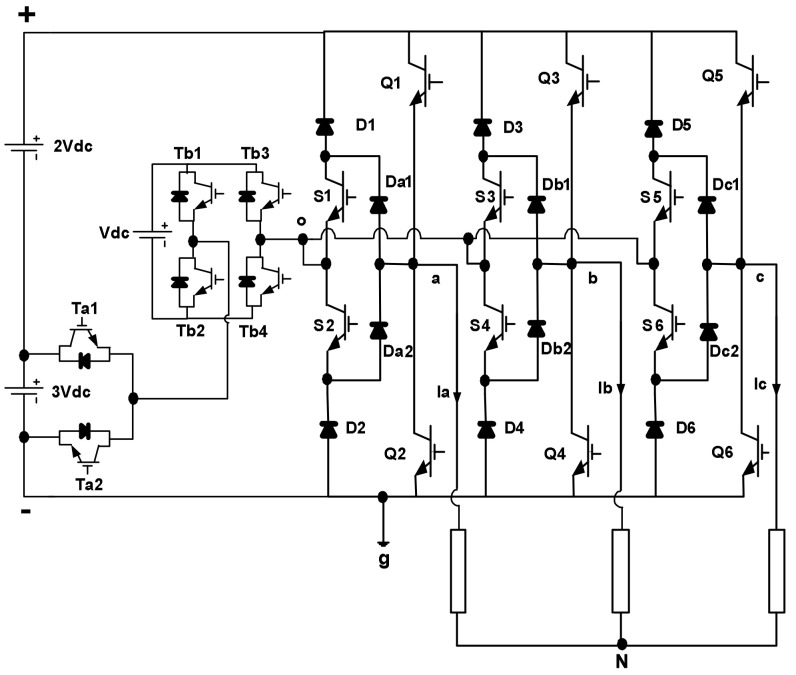
Proposed structure of a three-phase six-level inverter.

The circuit diagram of the proposed three-phase six-level MLI topology is depicted in Figure 1. Six unidirectional switches formed by (S1~S6) and (Da1~Dc2) diodes are connected with a classical three-phase six-switch (Q1~Q6) to build the main bridge. On the other hand, a single DC voltage supply, a half-bridge cell consisting of two switches (Ta1, Ta2) and a single DC supply along with a full-bridge power cell comprising four switches (Tb1~Tb4) and a DC supply are connected together to form a multilevel DC-link voltage. The DC voltages for half-bridge and full-bridge power cells are 3Vdc and Vdc, respectively, while the DC voltage for a single supply is 2Vdc. The power cell switches are turned on/off to synthesize four consecutive voltage levels: 4Vdc, 3Vdc, 2Vdc and Vdc at the middle point (o) relating to the inverter’s ground. Table 1 illustrates the switching operation of the proposed inverter.

It is worth mentioning that the switching states demonstrated in Table 1 are applicable for all three phases. For phase b and c, it should be Q3, Q4, S3, S4 and Q5, Q6, S5, S6, respectively instead of Q1, Q2, S1, S2. The switching configuration of the proposed MLI is given in the following equation:

(1) $$\left[\begin{array}{c} Vag\\ Vbg\\ Vcg \end{array}\right]=\frac{5Vdc}{N-1}\times \left[\begin{array}{c} Sa\\ Sb\\ Sc \end{array}\right]$$

where *Sa*, *Sb* and *Sc* are switching positions. *Vag*, *Vbg* and *Vcg* are the MLI’s line to ground voltages and *N* = 6 is the number of voltage levels. Since the topology employed for the proposed MLI is modeled to obtain the three-phase well-adjusted line to line output voltages producing the highest number of voltage levels, a suitable switching structure is needed for generating the MLI’s gate pulses. Consecutively, these switching states generate the gate pulses and drive the studied MLI under all modulation indexes. Different modulation techniques have been suggested to control the multilevel inverters. These modulation methods are categorized depending on the operating switching frequency [25]. The low frequency modulation method known as selective harmonic elimination (SHE-PWM) is usually employed to predefine and pre-calculate the optimal switching angles. To obtain a solution for the angles, a set of equations is normally solved offline using numerical methods [26,27]. The proposed MLI can be easily controlled, and the desired outputs can be simply achieved with regard to the MLI’s switching states *Sa*, *Sb* and *Sc*. Therefore, a new modulation method was recently suggested in [28] where each phase in the proposed inverter is controlled independently. For a given modulation index *Ma*, the inverter’s switching states *Sa*, *Sb* and *Sc* are determined with respect to the MLI’s line to ground reference voltages *Vag_ref*, *Vbg_ref* and *Vcg_ref*. The correlation among the MLI’s reference line to ground voltages and the respective switching sequences is:

(2) $$\left[\begin{array}{c} Sa\\ Sb\\ Sc \end{array}\right]=\left(\frac{N-1}{5Vdc}\times \left[\begin{array}{c} Vag\_ref\\ Vbg\_ref\\ Vcg\_ref \end{array}\right]\right)$$

(3) $$\left[\begin{array}{c} Vag\_ref\\ Vbg\_ref\\ Vcg\_ref \end{array}\right]=\frac{Ma\times 5Vdc}{2}\times \left[\begin{array}{c} \cos(\omega t)\\ \cos(\omega t-\frac{2\pi }{3})\\ \cos(\omega t+\frac{2\pi }{3}) \end{array}\right]+\frac{5Vdc}{2}\times \left[1-\frac{Ma}{6}\cos(3\omega t)\right]\times \left[\begin{array}{c} 1\\ 1\\ 1 \end{array}\right].$$

According to (3), the 3rd harmonic element is added to MLI’s line to ground reference voltage waveforms. Therefore, *Ma* can extend to 1.15 without instigating over modulation.

### 2.2. Extended Structure

**Figure 2:**
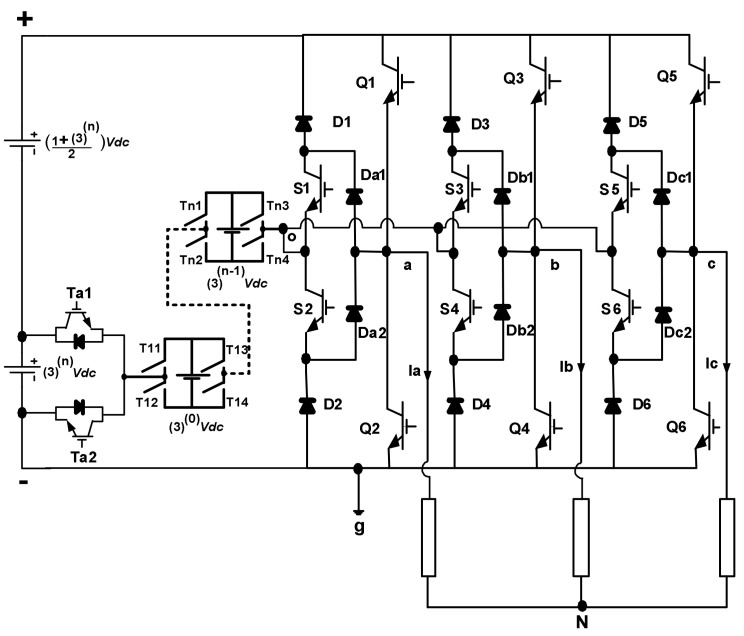
Proposed structure of three-phase *N*-level multilevel inverter.

The multistep DC-link circuit can be extended by keeping the proposed MLI’s main bridge construction. This enables the proposed inverter to achieve a higher amount of voltage steps using a small quantity of power equipment. To extend the multistep DC-link circuit, the half-bridge power module and (*n*) multiple units of full-bridge power modules are connected following series connection to generate (*N* – 1) sequent voltage steps as depicted in Figure 2. For full-bridge power cells:

(4) $$Vdc\_fb1=3^{\left(0\right)}Vdc$$

(5) $$Vdc\_fbn=3^{\left(n-1\right)}Vdc.$$

For half-bridge power cells:

(6) $$Vdc\_hb=3^{\left(n\right)}Vdc.$$

For a single DC voltage supply:

(7) $$Vdc\_s=Vdc+\sum_{i=1}^{i=n}3^{\left(n-1\right)}Vdc=\left(\frac{1+3^{\left(n\right)}}{2}\right)Vdc.$$

Hence, the highest number of voltage steps that can be achieved are:

(8) $$N=\frac{3}{2}\left(1+3^{\left(n\right)}\right).$$

Here, *N* = [6, 15, 42, 123, ........]. The total required numbers of IGBTs (*M_sw*) and DC supplies (*M_DC*) in terms of voltage steps are given as:

(9) $$M\_sw=4Log_{3}\left(\frac{2}{3}N-1\right)+14$$

(10) $$M\_DC=Log_{3}\left(\frac{2}{3}N-1\right)+2.$$

### 2.3. Efficiency Calculation

It is important to validate the two principal losses that generally occur during the operation of switching devices. These losses are classified as conduction losses and switching losses. Conduction losses (*P_Conduction_*) are defined as the losses of power electronic devices during on-state. In this case, both the diode and the switches are taken into consideration. The instantaneous conduction losses of the IGBT and diodes can be determined using (11) and (12), respectively.

*P_Conduction_IGBT_* (*t*) = [*V_IGBT_* + *R_IGBT_*
*i*(*t*)] × *i*(*t*) (11)

*P_Conduction_Diode_* (*t*) = [*V_Diode_* + *R_Diode_*
*i*(*t*)] × *i*(*t*) (12)

Here, *V_IGBT_* is the voltage of IGBTs while *V_Diode_* is the voltage of the diodes when these devices are activated. Again, *R_IGBT_* is the corresponding resistance of IGBTs while *R_Diode_* represents the corresponding resistance of the diodes. The subsequent results are then summed to calculate the conduction losses of the proposed MLI.

Switching losses (*P_Switching_*) are defined as the losses of power electronic modules when they are activated and deactivated. Switching loss is proportional to the switching frequency. The activation (*W_on_*) and deactivation (*W_off_*) energy loss of these modules are obtained by:

(13) $$W_{\mathrm{on}}\;=\;\overset{t_{\mathrm{on}}}{\underset{0}{\int }}\left[\left(\frac{v_{\mathrm{switch}}}{t_{\mathrm{on}}}t\right)\left(-\frac{I}{t_{\mathrm{on}}}(t-t_{\mathrm{on}}\right)\right]dt.$$

(14) $$W_{\mathrm{off}}=\overset{t_{\mathrm{off}}}{\underset{0}{\int }}\left[\left(\frac{v_{\mathrm{switch}}}{t_{\mathrm{off}}}t\right)\left(-\frac{I}{t_{\mathrm{off}}}(t-t_{\mathrm{off}}\right)\right]dt.$$

Here, *t_on_* and *t_off_* are the turn-on time and turn-off time of the IGBTs consecutively. *I* represents the current through the IGBT devices before/after they are turned off/on. *v*_switch_ demonstrates the forward voltage drop for the IGBTs. Thus,

(15) $$P_{\mathrm{switch}}=f\;\left[\overset{N_{\mathrm{switch}}}{\underset{i=1}{\sum }}\left(\overset{N_{\mathrm{on}}}{\underset{i=1}{\sum }}W_{\mathrm{on},i}+\overset{N_{\mathrm{off}}}{\underset{i=1}{\sum }}W_{\mathrm{off},i}\right)\right]$$

where *f*, *N_on_* and *N_off_* are the fundamental frequency and the number of times each switch is turned on and off during a time period of *t*, respectively. Again, *W_on_*_,*i*_ and *W_off_*_,*i*_ are the energy loss of each switch turning on and off for *i*th time. Thus, the total loss (*P_Total_*) can be calculated as follows:

*P_Total_* = *P_conduction_* + *P_switching._* (16)

The following parameters are considered for calculating the power loss of the proposed module: *V_IGBT_* = 2.4 V, *R_IGBT_* = 0.052 Ω, *V_Diode_* = 2 V and *R_Diode_* = 0.1 Ω, *f* = 50 Hz, *t_on_* = *t_off_* = 1. The proposed inverter is running under the conditions: Total DC-link voltage = 450 V, *Ma* = 1, *f* = 50 Hz, and *P*out = 3.6 kW. Again, a single-phase resistive inductive load (237 Ω˗0.53 H) is connected with the module as the output. The calculation is conducted for each switch and diode for one period and applying (11) to (16) the total losses (*P_Total_*) of the inverter is evaluated to be 88.75 W operating for 1 s. Therefore, the proposed inverter efficiency is 97.59%. This has passed the IEEE 1547 standard for interconnected devices of power grids.

### 2.4. Switching Pulses Generation Using Nearest Vector Modulation

**Figure 3:**
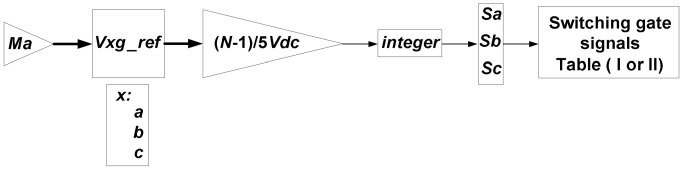
Operating principle of the NVM technique.

The goal of the modulation strategy is to produce the appropriate switching pulses by determining the MLI’s switching states. The operating block diagram of the nearest vector modulation (NVM) technique is shown in Figure 3. The space vector illustration of the operation of the projected MLI in stationary *α*−*β* location frame is obtained as follows:

(17) $$V\alpha =\frac{5Vdc}{3\left(N-1\right)}\times \left(2Sa-Sb-Sc\right)$$

(18) $$V\beta =\frac{5Vdc}{\sqrt{3}\left(N-1\right)}\times \left(Sc-Sb\right)$$

(19) V=Vα−jVβ.

**Figure 4:**
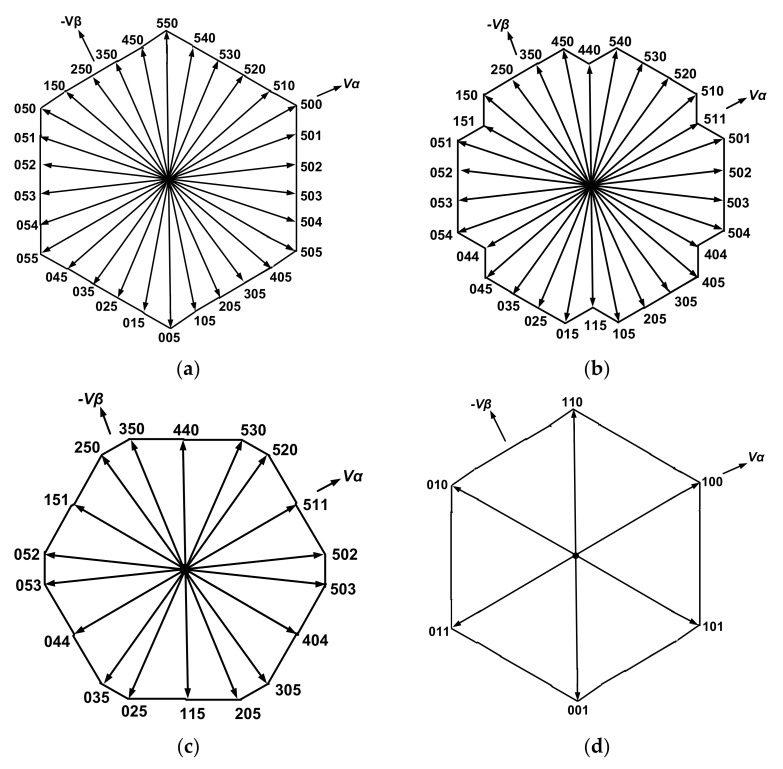
Space vector diagrams for the proposed MLI at: (**a**) *Ma* = 1.3, (**b**) *Ma* = 1.15, (**c**) *Ma* = 0.98 and (**d**) *Ma* = 0.8.

Four different space vector diagrams representing the function of the three-phase MLI with four valid switching sequences are shown in Figure 4.

## 3. Simulation Results

According to these space vector diagrams shown in Figure 4, the switching states of the proposed MLI can be classified into four categories.

(1) In case of *Ma* ≥ 1.2, the resultant switching state compromises of 30 various groupings of switching sequences (active vectors) are as follows: {055-054-053-052-051-050-150-250-350-450-550-540-530-520-510-500-501-502-503-504-505-405-305-205-105-005-015-025-035-045}.

**Figure 5:**
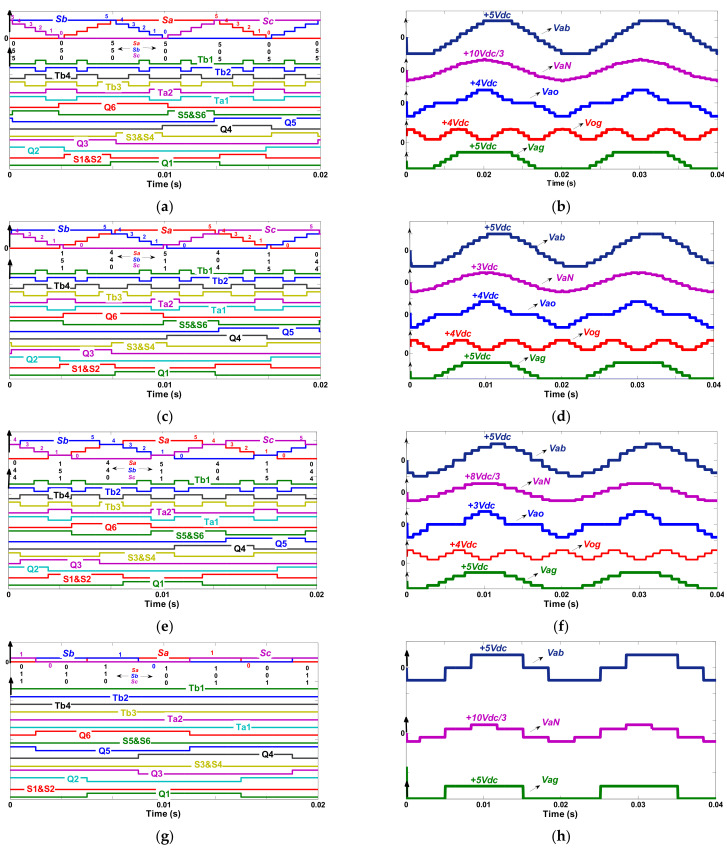
Simulation results: (**a**) switching states at *Ma* = 1.3, (**b**) simulated waveforms at *Ma* = 1.3 (**c**) switching states at *Ma* = 1.15, (**d**) simulated waveforms at *Ma* = 1.15, (**e**) switching states at *Ma* = 0.98, (**f**) simulated waveforms at *Ma* = 0.98, (**g**) switching states at *Ma* = 0.8 and (**h**) simulated waveforms *Ma* = 0.8.

For instance: at *Ma* = 1.3, Figure 5a shows the instantaneous values of *Sa*, *Sb* and *Sc* and switching pulses arrangements within a full cycle of operation. These switching states allow the proposed MLI to produce a three-phase stable staircase 11 level line to a line voltage with mutual variance of *Vdc*, which is as follows: 5*Vdc*, +4*Vdc*, +3*Vdc*, +2*Vdc*, +*Vdc*, 0, −*Vdc*, −2*Vdc*, −3*Vdc*, −4*Vdc*, −5*Vdc*) where *Vab*, *Vbc* and *Vca* are related to *Sa*, *Sb* and *Sc* by:

(20) $$\left[\begin{array}{c} Vab\\ Vbc\\ Vca \end{array}\right]=\frac{5Vdc}{N-1}\times \left[\begin{array}{ccc} 1 & -1 & 0\\ 0 & 1 & -1\\ -1 & 0 & 1 \end{array}\right]\times \left[\begin{array}{c} Sa\\ Sb\\ Sc \end{array}\right]$$

The simulated outputs including the MLI’s line to line (*V_ab_*), line to neutral (*V_aN_*), line to ground (*V_ag_*), line to center (*V_ao_*) and center to ground (*V_og_*) voltage outputs are illustrated in Figure 5b.

(2) For 1.2 > *Ma* > 0.98, the maneuver of the proposed MLI is organized inside 30 other combinations of switching sequences (active vectors) as follows: {044 (155)-054-053-052-051-151 (040)-150-250-350-450-440-(551)-540-530-520-510-511 (400)-501-502-503-504-404 (515)-405-305-205-105-115 (004)-015-025-035-045}.

The switching sequences of the proposed MLI and its equivalent switching pulses at (for instance: *Ma* = 1.15) are shown in Figure 5c. Reducing the modulation index affects the peak value voltage. Thus, the amount of line to neutral (*V_aN_*) voltage levels of the MLI, in this case, is decreased to 14 voltage levels, as shown in Figure 5d. These voltage levels are as follows: (+9*Vdc/*3, +8*Vdc/*3, +7*Vdc/*3, +6*Vdc/*3, +4*Vdc/*3, +3*Vdc/*3, +*Vdc/*3, −*Vdc/*3, −3*Vdc/*3, −4*Vdc/*3, −6*Vdc/*3, −7*Vdc/*3, −8*Vdc/*3 and −9*Vdc/*3). Although this switching category has decreased the peak voltage and the line to neutral voltage, the number of line to line voltage (*V_ab_*) outputs is maintained at 5*Vdc* with 11 voltage levels (+5*Vdc*, +4*Vdc*, +3*Vdc*, +2*Vdc*, +*Vdc*, *0*, −*Vdc*, −2*Vdc*, −3*Vdc*, −4*Vdc*, −5*Vdc*). 

From Figure 5d, it can be further observed that utilizing the synthesized switching states, six voltage steps can be generated using multiple combinations of switching sequences. For example, the voltage levels *Vab* = −4*Vdc*, *Vbc* = 0 and *Vca* = +4*Vdc* can be produced applying two valid redundant switching states 044 or (155). Since the switching sequence design should minimize the number of switching instances, the redundant switching states: {(155)-(040)-(551)-(400)-(515)-(004)} are not utilized.

(3) For *Ma* = 0.98, 18 different combinations of switching states (active vectors) are generated to form the new valid switching sequence which is as follows: {044 (155)-053-052-151 (040)-250-350-440 (551)-530-520-511 (400)-502-503-404 (515)-305-205-115 (004)-025-035}.

Figure 5e depicts the switching sequences of the projected MLI and switching pulses under the condition of *Ma* = 0.98. It can be observed that the MLI’s line to line voltage (*V_ab_*) waveforms reach their highest values of +5*Vdc* compromising nine voltage levels (+5*Vdc*, +4*Vdc*, +3*Vdc*, +2*Vdc*, *0*, −2*Vdc*, −3*Vdc*, −4*Vdc*, −5*Vdc*), while the inverter line to neutral voltages (*V_aN_*) reach their maximum value of +8*Vdc/*3 with eight voltage steps (+8*Vdc/*3, +7*Vdc/*3, +4*Vdc/*3, +*Vdc/*3, −*Vdc/*3, −4*Vdc/*3, +7*Vdc/*3, −8*Vdc/*3). It is worth mentioning that the operation under the condition of *Ma* = 0.98 makes the common voltage deference in staircase waveform of nine line to line voltage levels fluctuate between *Vdc* and 2*Vdc*. As a result, the MLI functions like a three-phase five-level asymmetrical MLI. 

The simulated staircase waveforms of voltages at *Ma* = 0.98 are shown in Figure 5f. Similar to the previous case, the six redundant switching states in this switching sequence: {(155)-(040)-(551)-(400)-(515)-(004)} are also not utilized.

(4) For *Ma* < 0.98, the MLI functions like a traditional three-phase two-level MLI. The novel switching sequences are demonstrated in Table 2 (For instance: at *Ma* = 0.8). The switching sequences tabulated in Table 2 are also applicable for other phases. 

In this case, the MLI’s switching pulses are generated utilizing the switching sequence shown in Figure 5g. It is identified that the final switching state comprises of six groupings of active vectors: {011-010-110-100-101-001}. Following this switching state leads the projected MLI to obtain the desired output voltage of *Vab*, *VaN* and *Vag* as depicted in Figure 5h.

**Figure 6:**
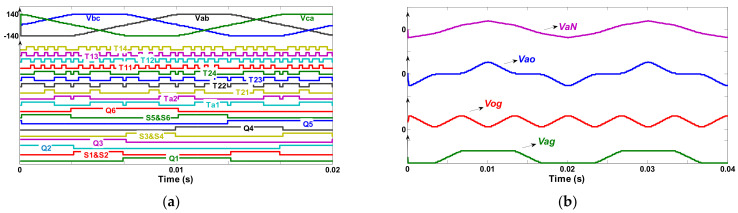
For (*n* = 2, *N* = 15, *f* = 50 Hz) (**a**) Switching gate signals and *Vab*, *Vbc* and *Vca*, (**b**) Simulated waveforms of *VaN*, *Vao*, *Vog* and *Vag*.

The extended configuration is also verified by simulating a model for (*n* = 2). The simulation is done utilizing 10 V voltage supply and a three-phase resistive-inductive load of 237 Ω–0.53 H as output. The nearest vector modulation technique is implemented with a nominal frequency of 50 Hz. The simulation results of fifteen-level line to line output voltage (*V_ab_*) built on the extended model and its equivalent switching pulses are illustrated in Figure 6a. In addition, the output voltages of *VaN*, *Vao*, *Vog* and *Vag* are also shown in Figure 6b.

## 4. Comparative Analysis

**Figure 7:**
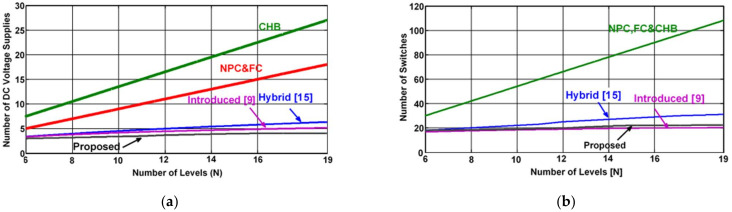
Comparison in terms of: (**a**) DC supplies, (**b**) switches.

The major advantage of the proposed MLI configuration is the ability to generate a high amount of voltage steps utilizing a small number of power electric components, as depicted in Table 3 and Figure 7. To efficiently achieve the required output voltage and current, the investigated MLI requires appropriate power devices. Thus, it is necessary to determine their rated power, voltage and current. Since the proposed inverter is built by a multilevel DC-link and three-phase Bridge, it uses switches with different voltage ratings, as illustrated in Table 4. Furthermore, it was assumed that all power devices of the inverter have the same current rating. Compared to other multilevel inverters listed in Table 5, the proposed inverter requires high voltage rating switches. As a result of this comparison, the total average cost per switch for the proposed inverter is higher than other NPC, FC, CHB and hybrid multilevel inverter switches, and approximately equal to that calculated in [29,30]. This additional cost can be mostly compensated for, since the proposed inverter requires a lower amount of DC supplies, IGBTs, diodes and gate drivers. In general, when matched by additional MLI structures, the proposed MLI’s major benefits are: (1) a higher number of voltage levels applying power components in small numbers, (2) a low-cost heat sink, smaller installation area and low gate drivers being required, (3) inheriting certain benefits of traditional two-level inverters such as a lesser number of control signals, simple operating standard and low conduction loss. It also has similar advantages to multilevel inverters such as reduced switching losses, small harmonic distortions and improved performance. In contrast, the disadvantages are: (1) IGBT switches (Q1~Q6) must sustain the complete DC-link voltage once the MLI generates the maximum voltage ±5*Vdc*, whereas the bidirectional, half-bridge and full-bridge IGBTs required to sustain ±4*Vdc*, ±3*Vdc* and ±*Vdc*, respectively. As a result, different power ratings on different types of switches are required for building the proposed inverter. (2) Voltage levels (six) in maximum number can only be obtained when the modulation index is greater than 0.98, otherwise the behavior of the MLI becomes akin to a classical two-level inverter. Therefore, the projected MLI is highly appropriate for PV applications working on medium voltage conditions [31] and following fundamental switching frequency at the fixed modulation index Ma > 0.98. 

## 5. Experimental Validation and Results

### 5.1. Experimental Setup 

**Figure 8:**
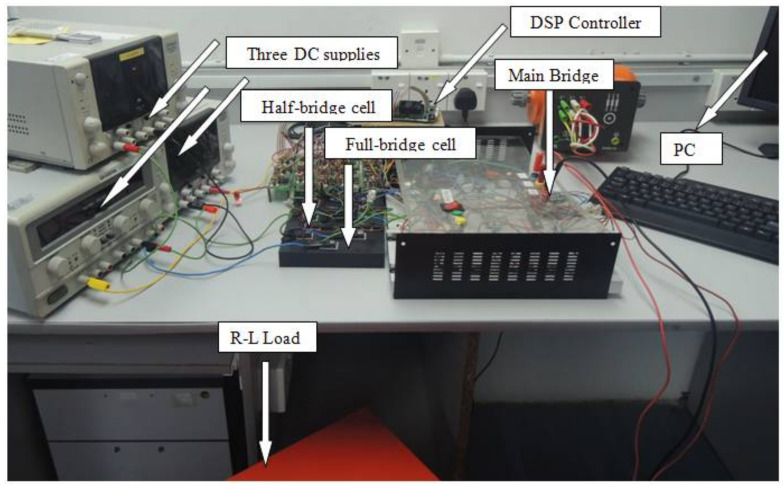
Laboratory setup of the hardware prototype.

**Figure 9:**
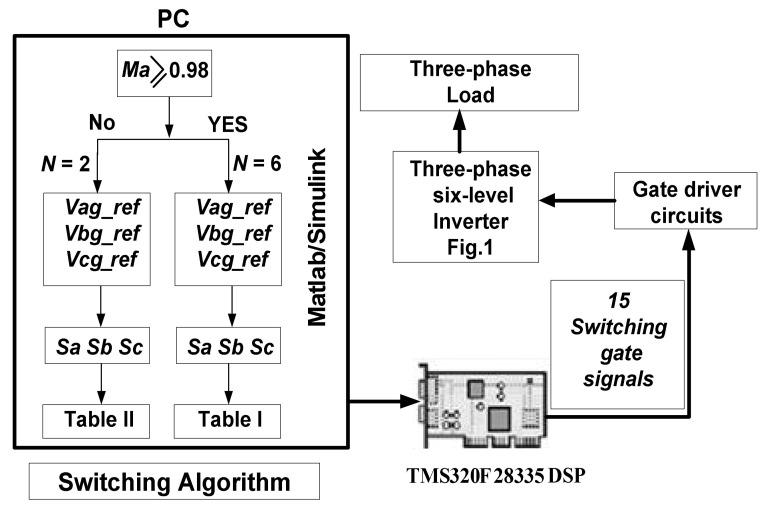
Control block diagram.

An inverter prototype is built and tested by applying *f* = 50 Hz and *Ma* = (1.3, 1.15, 0.98 and 0.8) for validating the compatibility of the studied modulation method and the performance of the proposed inverter, as shown in Figure 8. Three isolated DC voltage supplies having different magnitudes (*Vdc* = 20 V, 3*Vdc* = 60 V and 2*Vdc* = 40 V) are used to form the multilevel DC-link. A three-phase load (237 Ω–0.5 H) is used as the output. The inverter control algorithm based on the suggested modulation technique is developed using Simulink/ MATLAB software and TMS320F28335 DSP controller. Figure 9 illustrates the proposed MLI’s control diagram.

### 5.2. Experimental Results 

**Figure 10:**
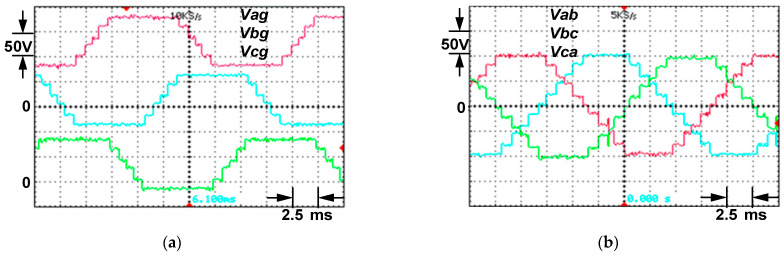

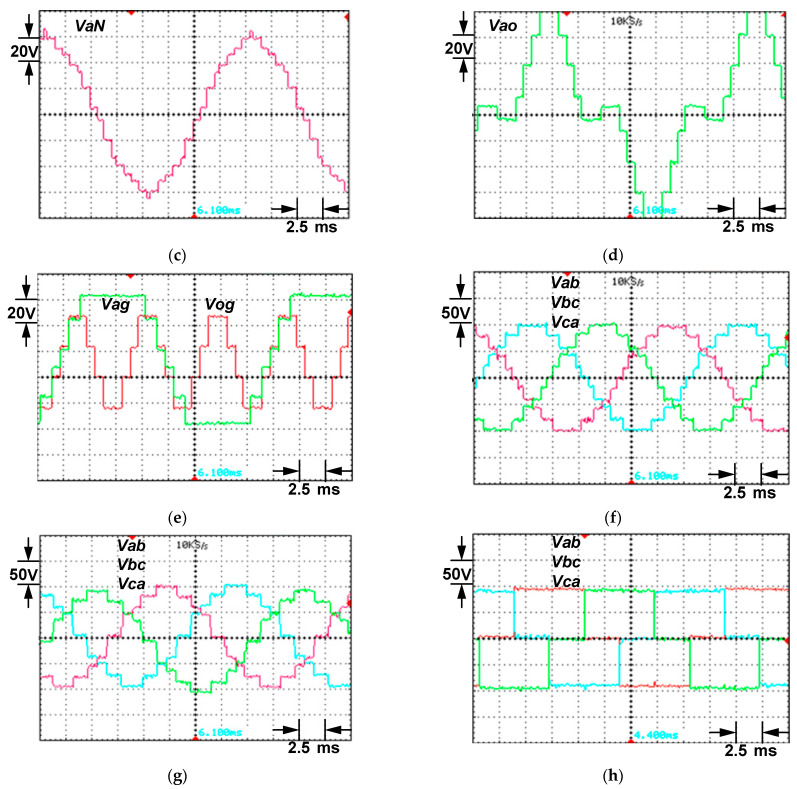
For *Ma* =1.3, the MLIs are: (**a**) line to ground voltages, (**b**) line to line voltages, (**c**) line to neutral voltage, (**d**) line to mid-point voltage and (**e**) line to ground and mid-point ground voltages, (**f**) For *Ma* =1.15, line to line voltages, (**g**) For *Ma* = 0.98, line to line voltages, (**h**) For *Ma* = 0.8, line to line voltages.

The inverter line to ground, line to line, line to neutral, line to center and center to ground outputs are shown in Figure 10. For *Ma* = 1.3, the waveforms for *Vag*, *Vbg* and *Vcg* have six symmetric voltage levels with the highest value of 100 V, as shown in Figure 10a. It should be addressed that the experimental results are presented following the RGB color format, which means all the waveforms of phase *a* are represented by red color, while the waveforms of phase *b* and phase *c* are represented by a green and blue color, respectively. 

**Figure 11:**
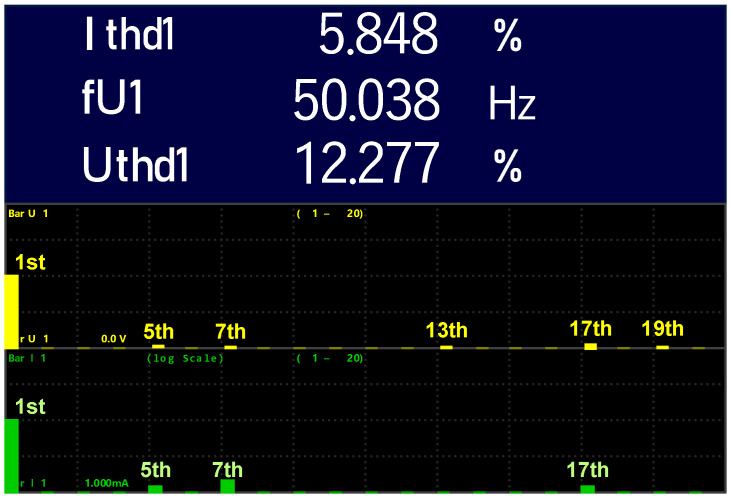
Harmonic spectrum of load current (yellow) and line to line voltage (green).

The waveforms of *Vab*, *Vbc* and *Vca* are composed of 11 voltage steps with a peak value of 100 V, as depicted by Figure 10b. In Figure 10c, the inverter line to neutral *VaN* reached its peak value of 63 V with 16 voltage steps, while the waveform of *Vao* attained the peak voltage of 80 V producing nine voltage levels as shown in Figure 10d. From Figure 10e, four different voltage values are taken from inverter mid-point to ground *Vog*, with the peak value of 80 V. Then, within a full cycle of *Vag*, a repetition occurs three times. In Figure 10f,g, the proposed MLI’s line to line output voltage at modulation index, *Ma* = 1.15 and 0.98 are depicted, respectively. The performance of the proposed MLI becomes the same as that of a traditional two-level inverter when *Ma* = 0.8, as shown in Figure 10h. The harmonic spectrum for the waveforms of inverter line to line voltage and load current at *Ma* = 1 are depicted in Figure 11.

According to Figure 11, both spectrums do not contain any triplen harmonics such as 3rd, 9th and 15th harmonics. Furthermore, all even harmonics are eliminated because the MLI’s output voltage and load current have obtained symmetry. %THD for the load current is only 5.8%, while the THD for MLI’s line to line voltage is 12.3%.

## 6. Conclusions

A novel three-phase six-level multilevel inverter structure was developed and is outlined in this manuscript. The designed MLI possesses some advantageous characteristics topology such as it requires the least amount of power components and DC supplies, the prototype can be built-in cost-effective manner and it can be controlled using a simple modulation technique. A new approach based on the nearest vector modulation technique was easily implemented and the switching gate signals were successfully generated. Furthermore, a three-phase *N*-level structure based on the proposed configuration was suggested and discussed. The proposed MLI validity and suggested modulation technique compatibility was validated by employing both experimental and simulation approaches. The simple and pragmatic design of the proposed MLI would make it a highly suitable candidate for medium voltage applications in distributed renewable energy applications and grid-connected systems. The low current THD and higher efficiency generated by the MLI will allow it to be utilized in various industrial applications. The concluding remarks of the manuscript can be listed as follows:

1. The proposed MLI utilized a reduced number of power electric components compared to classical and other recently developed MLIs.
2. The modularity of the MLI makes it a suitable candidate for high voltage operations.
3. The operating efficiency of the MLI is 97.59% and effectively follows the IEEE 1547 standard, making it suitable to be applied as an interconnected device in grid systems.
4. The low current THD of 5.8% would allow this inverter to be utilized in the grid’s current and voltage compensation systems, including shunt active power filters (SAPFs), voltage restorers and unified power quality conditioners (UPQCs).

## Figures and Tables

**Table 1:** Switching states for the proposed six-level inverter (*Ma* ≥ 0.98).

Sa	Q1	S1	S2	Q2	Ta1	Ta2	Tb1	Tb2	Tb3	Tb4	Vag
5	1	0	0	0	0	0	0	0	0	0	+5*Vdc*
4	0	1	1	0	1	0	0	1	1	0	+4*Vdc*
3	0	1	1	0	1	0	0	1	0	1	+3*Vdc*
2	0	1	1	0	1	0	1	0	0	1	+2*Vdc*
1	0	1	1	0	0	1	0	1	1	0	+*Vdc*
0	0	0	0	1	0	0	0	0	0	0	0

**Table 2:** Switching states for the proposed six-level inverter (*Ma* < 0.98).

Sa	Q1	S1	S2	Q2	Ta1	Ta2	Tb1	Tb2	Tb3	Tb4	Vag
1	1	0	0	0	0	0	0	0	0	0	+5*Vdc*
0	0	0	0	1	0	0	0	0	0	0	0

**Table 3:** Comparative analysis between different multilevel inverter topologies in terms of voltage levels.

Components	NPC	FC	CHB	[9]	[15]	Proposed
Switches	6 (*N* − 1)	6 (*N* − 1)	6 (*N* − 1)	2 log_2_ (*N* − 1) + 12	12 [log_3_ (*N*/2) − 1] + 18	4 log_3_ [2/3 (*N*) − 1] + 14
Diodes	6 (*N* − 1)	6 (*N* − 1)	6 (*N* − 1)	2 log_2_ (*N* − 1) + 12	12 [log_3_ (*N*/2) − 1] + 18	4 log_3_ [2/3 (*N*) − 1] + 14
Clamping diodes	6 (*N* − 2)	0	0	0	0	0
Gate drivers	6 (*N* − 1)	6 (*N* − 1)	6 (*N* − 1)	2 log_2_ (*N* − 1) + 9	12 [log_3_ (*N*/2) − 1] + 18	4 log_3_ [2/3 (*N*) − 1] + 11
DC supplies	*N* − 1	*N* – 1	3 (*N* − 1)/2	1 + log_2_(*N* − 1)	3 [log_3_ (*N*/2) − 1] + 4	log_3_ [2/3 (*N*) − 1] + 2
Capacitors	0	3 (*N* − 2)	0	0	0	0
Control signals	6 (*N* − 1)	6 (*N* − 1)	6 (*N* − 1)	2 log_2_ (*N* − 1) + 9	12 [log_3_ (*N*/2) − 1] + 18	4 log_3_ [2/3 (*N*) − 1]+11

**Table 4:** Voltage ratings of proposed topology switches.

Switches	Main Bridge	Bidirectional	Half-Bridge	Full-Bridge
Voltage rating	(*N* − 1) *Vdc*	(*N* − 2) *Vdc*	3*^n^* (*Vdc*)	3^(*n* − 1)^ (*Vdc*)

**Table 5:** Voltage rating of other multilevel inverter topologies.

Voltage Ratings	NPC	FC	CHB	[9]	[15]
Switch	*Vdc*	*Vdc*	*Vdc*	(*N* − 1) *Vdc*	*Vdc*
Diode	*Vdc*	0	0	0	0
Capacitor	0	*Vdc*	0	0	0

## Data Availability

Not applicable.

## References

[1] Shayestegan M., Shakeri M., Abunima H., Reza S.S., Akhtaruzzaman M., Bais B., Mat S., Sopian K., Amin N. (2018). An overview on prospects of new generation single-phase transformerless inverters for grid-connected photovoltaic (PV) systems. Renew. Sustain. Energy Rev..

[2] Yuan X. (2014). A set of multilevel modular medium-voltage high power converters for 10-MW wind turbines. IEEE Trans. Sustain. Energy.

[3] Meraj S.T., Hasan M.K., Islam J., El-Ebiary Y.A.B., Nebhen J., Hossain M., Alam K., Vo N. (2021). A diamond shaped multilevel inverter with dual mode of operation. IEEE Access.

[4] Hamidi M.N., Ishak D., Zainuri M.A.A.M., Ooi C.A. (2020). An asymmetrical multilevel inverter with optimum number of components based on new basic structure for photovoltaic renewable energy system. Sol. Energy.

[5] Hannan M.A., Ali J.A., Mohamed A., Uddin M.N. (2016). A random forest regression based space vector PWM inverter controller for the induction motor drive. IEEE Trans. Ind. Electron..

[6] Law K.H. (2018). An effective voltage controller for quasi-Z-source inverter-based STATCOM with constant DC-link voltage. IEEE Trans. Power Electron..

[7] Jibhakate C., Chaudhari M., Renge M. (2018). A reduced switch AC–AC converter with the application of D-STATCOM and induction motor drive. Electronics.

[8] Lee J., Kang D., Lee J. (2019). A study on the improved capacitor voltage balancing method for modular multilevel converter based on hardware-in-the-loop simulation. Electronics.

[9] Katebi R., He J., Weise N. (2018). An advanced three-level active neutral-point-clamped converter with improved fault-tolerant capabilities. IEEE Trans. Power Electron..

[10] Nair V., Rahul A., Kaarthik S., Kshirsagar A., Gopakumar K. (2017). Generation of higher number of voltage levels by stacking inverters of lower multilevel structures with low voltage devices for drives. IEEE Trans. Power Electron..

[11] Samadaei E., Kaviani M., Iranian M., Pouresmaeil E. (2019). The P-type module with virtual DC links to increase levels in multilevel inverters. Electronics.

[12] Meraj S.T., Yahaya N.Z., Hasan K., Masaoud A. (2021). A hybrid T-type (HT-type) multilevel inverter with reduced components. Ain Shams Eng. J..

[13] Mahato B., Mittal S., Majumdar S., Jana K., Nayak P.K. (2018). Multilevel inverter with optimal reduction of power semi-conductor switches. Renewable Energy and Its Innovative Technologies.

[14] Zeng J., Lin W., Cen D., Liu J. (2020). Novel K-type multilevel inverter with reduced components and self-balance. IEEE J. Emerg. Sel. Top. Power Electron..

[15] Gautam S.P., Kumar L., Gupta S. (2015). Hybrid topology of symmetrical multilevel inverter using less number of devices. IET Power Electron..

[16] Vijayarajan P., Shunmugalatha A., Sathik J. (2017). A new hybrid multilevel inverter topology for medium and high voltage applications. Appl. Math. Inf. Sci..

[17] Babaei E., Laali S., Alilu S. (2014). Cascaded multilevel inverter with series connection of novel h-bridge basic units. IEEE Trans. Ind. Electron..

[18] Masaoud A., Mekhilef S., Ping H.W., Wong K.I. (2017). A simplified structure for three-phase 4-level inverter employing fundamental frequency switching technique. IET Power Electron..

[19] Raushan R., Mahato B., Jana K.C. (2016). Comprehensive analysis of a novel three-phase multilevel inverter with minimum number of switches. IET Power Electron..

[20] Hota A., Jain S., Agarwal V. (2017). An optimized three-phase multilevel inverter topology with separate level and phase sequence generation part. IEEE Trans. Power Electron..

[21] Norambuena M., Kouro S., Dieckerhoff S., Rodriguez J. (2018). Reduced multilevel converter: A novel multilevel converter with a reduced number of active switches. IEEE Trans. Ind. Electron..

[22] Thiyagarajan V., Somasundaram P., Kumar K.R. (2019). Simulation and analysis of novel extendable multilevel inverter topology. J. Circuits Syst. Comput..

[23] Hamidi M.N., Ishak D., Zainuri M.A.A.M., Ooi C.A. (2020). Multilevel inverter with improved basic unit structure for symmetric and asymmetric source configuration. IET Power Electron..

[24] Meraj S.T., Yahaya N.Z., Hasan K., Masaoud A. (2020). Single phase 21 level hybrid multilevel inverter with reduced power components employing low frequency modulation technique. Int. J. Power Electron. Drive Syst..

[25] Rodriguez J., Lai J.-S., Peng F.Z. (2002). Multilevel inverters: A survey of topologies, controls, and applications. IEEE Trans. Ind. Electron..

[26] Ahmadi D., Wang J. (2013). Online selective harmonic compensation and power generation with distributed energy resources. IEEE Trans. Power Electron..

[27] Islam J., Meraj S.T., Masaoud A., Mahmud A., Nazir A., Kabir M.A., Hossain M., Mumtaz F. (2021). Opposition-based quantum bat algorithm to eliminate lower-order harmonics of multilevel inverters. IEEE Access.

[28] Oskouei A.B., Dehghanzadeh A.R. (2015). Generalized space vector controls for MLZSI. Ain Shams Eng. J..

[29] Sayago J.A., Bruckner T., Bernet S. (2008). How to select the system voltage of mv drives—A comparison of semiconductor expenses. IEEE Trans. Ind. Electron..

[30] Luo J., Lin K., Li J., Xue Y., Zhang X.-P. Cost analysis and comparison between modular multilevel converter (MMC) and modular multilevel matrix converter (M3C) for offshore wind power transmission. Proceedings of the 15th IET International Conference on AC and DC Power Transmission (ACDC 2019).

[31] Wang Z., Li G., Tseng M.-L., Wong W.-P., Liu B. (2020). Distributed systematic grid-connected inverter using IGBT junction temperature predictive control method: An optimization approach. Symmetry.

